# Association of the human gut microbiota with vascular stiffness

**DOI:** 10.1038/s41598-023-40178-6

**Published:** 2023-08-16

**Authors:** Rafael R. C. Cuadrat, Tobias Goris, Anna Birukov, Fabian Eichelmann, Bruno G. N. Andrade, Corinna Bang, Andre Franke, Clemens Wittenbecher, Matthias B. Schulze

**Affiliations:** 1 Department of Molecular Epidemiology, German Institute of Human Nutrition Potsdam-Rehbruecke, Arthur-Scheunert-Allee 114-116, 14558 Nuthetal, Germany; 2 German Center for Diabetes Research (DZD), München-Neuherberg, Germany; 3 Bioinformatics and Omics Data Science, Berlin Institute for Medical Systems Biology (BIMSB), Max Delbrück Center (MDC), Berlin, Germany; 4 Research Group Intestinal Microbiology, Department of Molecular Toxicology, German Institute of Human Nutrition Potsdam-Rehbruecke, Arthur-Scheunert-Allee 114-116, 14558 Nuthetal, Germany; 5 Department of Computer Science, Munster Technological University, MTU/ADAPT, Cork, Ireland; 6 Institute of Clinical Molecular Biology, Christian-Albrechts-University of Kiel, Kiel, Germany; 7 Department of Nutrition, Harvard T.H. Chan School of Public Health, Boston, MA USA; 8 Institute of Nutritional Science, University of Potsdam, Nuthetal, Germany

**Keywords:** Risk factors, Cardiovascular diseases

## Abstract

Gut microbiota metabolites have been mechanistically linked to inflammatory pathway activation and atherosclerosis, which are major causes of vascular stiffness (VS). Aiming to investigate if the gut microbiome might be involved in VS development, we performed a cross-sectional study (n = 3,087), nested within the population-based European Prospective Investigations into Cancer and Nutrition (EPIC) Potsdam. We investigated the correlation of the gut microbiota (alpha diversity and taxa abundance) with 3 vascular stiffness measures: carotid-femoral (PWV), aortic augmentation index (AIX) and ankle-brachial index (ABI). Shannon index was not significantly associated with VS but the number of observed Amplicon Sequence Variants (ASV) was positively associated with PWV and AIX. We found a total of 19 ASVs significantly associated with at least one VS measure in multivariable-adjusted models. One ASV (classified as *Sutterella wadsworthensis*) was associated with 2 VS measures, AIX (− 0.11 ± 0.04) and PWV (-0.14 ± 0.03). Other examples of ASVs associated with VS were *Collinsella aerofaciens,* previously reported to be affected by diet and *Bacteroides uniformis,* commercially *available* as probiotics. In conclusion, our study suggests a potential role of individual components of the gut microbiota in the aetiology of VS.

## Introduction

Differences in the human gut microbiota have been associated with several diseases and general health status, such as type 2 diabetes (T2D)^1–3^, obesity^4,5^, hypertension^6–8^, and coronary artery disease (CAD)^9–11^. Animal models suggest causal links between the gut microbiome and cardiometabolic disease aetiology. For example, the susceptibility to atherosclerosis was suggested to be transferable by gut microbiota transplantation in mice^12^. Several mechanisms were proposed to explain the influence of the microbiome on the health status of its host. Some are direct effects, such as the microbial production of bile acids, coprostanol, ferulic acid (8,9) and trimethylamine-n-oxide (TMAO)^13^ which might lead to hypercholesterolemia and cardiovascular disease. There are also indirect mechanisms proposed; for example, the gut microbiome can modulate inflammatory pathways potentially leading to atherosclerosis and CAD^14^. Inflammation, atherosclerosis and ageing are major causes for arterial stiffness^15^, an independent predictor of cardiovascular disease risk^16^ that can cause systolic hypertension and promote left ventricular remodelling, dysfunction and failure^17^. Because of the established link between the gut microbiome and the major causes of vascular stiffness (VS), it was previously hypothesised that the gut microbiome can affect vascular health. So far, few studies were conducted to investigate this link^18,19^. These studies were relatively small and focused on the carotid-femoral pulse wave velocity (PWV) or ambulatory arterial stiffness index (AASI) as indicators of VS which reflects large artery stiffness but not systolic wave reflection or peripheral artery disease (PAD), measured by aortic augmentation index (AIX) and ankle-brachial index (ABI), respectively. Whereas the study on the TwinsUK cohort found a significant negative correlation of the gut microbial diversity with VS^18^, a later study delivered contradicting results with no influence of diversity on VS^19^. Instead, this study found decreased short-chain fatty acids in plasma of individuals with VS. We therefore performed a larger cross-sectional study among 3,087 individuals, including both sexes, which was nested within the population-based European Prospective Investigations into Cancer and Nutrition (EPIC) Potsdam cohort, to investigate the correlation of the microbiota with a comprehensive set of vascular stiffness measures also including AIX and ABI.

## Methods

### Study design and participants

We performed a cross-sectional analysis within the EPIC-Potsdam Study, a population-based unselected longitudinal cohort from the municipality of Potsdam, Germany. At baseline, approx. 27,500 men and women were recruited between 1994 and 1998^20^. Participants were followed up via questionnaires regarding incident diseases, all-cause mortality, and anthropometric measures. Of those participants who responded to the 6^th^ wave of follow-up questionnaires between 2014 and 2016 (n = 15,424), n = 8,517 were randomly invited for a physical examination between 2014 and 2020 to collect data on nutritional and cardiovascular phenotyping and to identify risk factors for the development and progression of chronic diseases, such as T2D and cardiovascular diseases. Until 31st January 2020, 4,370 participants were physically examined in this substudy (EPIC-DZD). The set of analytical samples comprised 3,099 participants with available faecal samples and vascular stiffness measurements. All examinations were performed in quiet and temperature-controlled rooms by trained study nurses using standardised protocols. The study was conducted according to the Declaration of Helsinki and approved by the Ethical Committee of the State of Brandenburg, Germany (S9/2002). All participants provided written informed consent.

### Measurements of vascular stiffness

Parameters of vascular stiffness (carotid-femoral PWV, ABI) were recorded with Vascular Explorer (Enverdis GmbH, Jena, Germany). Vascular Explorer implements single-point, supra systolic brachial oscillometry pulse wave analysis for the assessment of PWV and AIX, and was validated against other oscillometric, photoplethysmographic and Doppler devices^21,22^. PWV is a proxy for large artery stiffness, reflecting arteriosclerosis, AIX is a measure of wave reflection aiming to quantify the effect of systolic wave reflection on cardiac workload and ABI (lower than 0.9) is an indicator of PAD^23^. Measurements were performed with appropriate arm and leg cuffs after at least 10 min rest in the supine position. Pulse wave analysis, ankle and brachial blood pressures were automatically computed by software mediated analysis of photoplethysmographic signals from finger and toe and volume changes in the inflatable cuffs. The ABI was calculated by the software using the blood pressure values in the lower and upper extremities. Participants were asked to refrain from speaking and encouraged to breathe calmly during the measurements. Exclusion criteria for this examination were amputations of both limbs, open wounds at measurement sites, dialysis shunts, paralyses, lymphedema on arms or legs, bandages, or anti-embolism compression stockings which could not be removed.

### Microbiome analysis

Participants self-collected stool samples in tubes with a DNA stabiliser (Stratec Molecular Stool collection tube) at home and sent the samples via mail back to the study centre, where the samples were transferred to a − 80° freezer until further processing. Median time from sampling to freezing was 2 days (interquartile range 1–3 days). Participants with acute or chronic intestinal diseases (including intestinal tumours) or those treated with antibiotics in the last 3 months were excluded from stool sampling.

Total DNA from stool samples was extracted using the QIAamp DNA fast stool mini kit automated on the QIAcube. Approximately 200 mg faecal slurry was transferred to 0.70 mm Garnet Bead tubes filled with 1 ml InhibitEx buffer. Subsequently, bead beating was performed using a SpeedMill PLUS for 45 s at 50 Hz. Samples were then heated to 95° C for 5 min and centrifuged afterwards for 1 min at 10,000 RPM. 200 µl of the resulting supernatant were transferred to a 2 ml microcentrifuge tube, which was placed in the QIAcube for follow-up automated DNA isolation according to the manufacturer’s protocol. Hypervariable regions V3 and V4 of the 16S rRNA gene were amplified using the primer pair 357F-806R in a dual-barcoding approach according to Kozich et al.^24^. DNA samples were diluted 1:10 prior PCR, and 3 µl of this dilution were finally used for amplification. PCR products were normalised using the SequalPrep Normalization Plate Kit (Thermo Fisher Scientific, Waltham, MA, USA), pooled equimolar and sequenced on the Illumina MiSeq v3 2 × 300 bp (Illumina Inc., San Diego, CA, USA).

The resulting fastq files were quality checked with FASTQC, cutadapt was used for removal of primer and adaptor sequences, and then the sequences were processed with the DADA2 package (v. 1.14.1) on R (v. 3.6.3) to generate Amplicon Sequence Variants (ASVs) and to exclude chimeric sequences. Bacterial sequences were taxonomically classified using SILVA (v. 132) whenever possible until species taxonomic rank. Alpha diversity metrics (Shannon, Simpson and Observed ASVs) were calculated based on rarefied samples (10.000 reads) using the R package phyloseq (v. 1.30.0).

### Statistical analyses

Alpha diversity metrics were adjusted for batch and sequencing run information using linear models (R base LM method). The residuals obtained from the model were standardized and used as the dependent variable in new linear models for each of vascular stiffness measures, as independent variables, adjusting for sex, age, mean arterial pressure (MAP) and body mass index (BMI).

ASVs were centred log-transformed (CLR) using the R package microbiome (v. 1.8.0) and a distance matrix (Euclidean distance), based on CLR transformed ASV was calculated using the phyloseq package. A Permutational Multivariate Analysis Of Variance (PERMANOVA) was performed using the Euclidian distance matrix with the R package vegan (v. 2.5–6) to check for associations with VS measures, adjusting for age, sex, MAP, shipping batch and sequencing batch. ASVs were then filtered by prevalence (keeping those in ≥ 20% of samples and with ≥ 0.1% of relative abundance), and individually tested for association with VS using the Microbiome Multivariable Association with Linear Models (Maaslin2) (v. 1.4), adjusting for (i) sex, age, MAP, BMI, batch and sequencing run (model A); (ii) the same of (i) with the addition of T2D status (model B). Additionally, we ran an effect measure modification test with each VS parameter, sex and T2D status. For that, we created an interaction term for each VS measure with T2D and sex and ran Maaslin2 with the same adjustments for model B. For all models, the p-values were adjusted for multiple testing using the Benjamini–Hochberg method and results with q-value ≤ 0.1 were considered for subsequent analyses and discussions. We also performed stratified analysis by the variable that VS interacted with (sex or prevalent T2D) for the ASVs showing significant associations with the interactions.

## Results

Participants’ characteristics stratified by sex are shown in Table 1. Of the participants, 57.1% were women, and for PWV and AIX, the average values were higher, and for ABI the values were lower in women. Our cohort included 688 participants with prevalent T2D (22.3%) and the average age was 68.6 years (SD 8.0).

**Table 1:** Participants’ characteristics of the EPIC-DZD study.

Characteristic	Female (N = 1,764)	Male (N = 1,323)	Total (N = 3,087)
Age (years)	67.0 (8.3)	70.8 (7.1)	68.6 (8.0)
Smoking			
Never, n (%)	1081 (61)	534 (40)	1615 (52)
Ex, n (%)	549 (31)	678 (51)	1227 (40)
Current, n (%)	134 (8)	111 (8)	245 (8)
BMI (kg/m^2^)	26.5 (4.8)	27.6 (4.0)	27.0 (4.5)
Systolic BP (mmHg)	138 (18)	143 (17)	140 (18)
Diastolic BP (mmHg)	80 (9)	80 (9)	80 (9)
HbA1c (%)	5.62 (0.61)	5.90 (0.86)	5.74 (0.74)
HDL-C (mmol/L)	1.69 (0.41)	1.35 (0.32)	1.54 (0.41)
LDL-C (mmol/L)	3.58 (0.96)	3.14 (1.00)	3.39 (1.00)
Triglycerides (mmol/L)	1.44 (0.75)	1.71 (1.03)	1.56 (0.89)
AST (µmol/s*l)	0.37 (0.23)	0.46 (0.24)	0.41 (0.24)
ALT (µmol/s*l)	0.41 (0.13)	0.45 (0.16)	0.43 (0.14)
T2D			
Yes, n (%)	263 (14.9)	425 (32.1)	688 (22.3)
No, n (%)	1,501 (85.1)	898 (67.9)	2,399 (77.7)
Medication			
Antidiabetic, n (%)	194 (11)	348 (26)	542 (18)
Antihypertensive, n (%)	851 (48)	827 (63)	1678 (54)
Lipid-lowering, n (%)	344 (20)	525 (40)	869 (28)
MAP (mmHg)	99.6 (11.0)	101.6 (10.8)	100.4 (11.0)
PWV (m/s)	9.77 (2.26)	9.16 (2.32)	9.51 (2.30)
AIX (%)	31.7 (9.7)	24.7 (10.9)	28.7 (10.8)
ABI	1.13 (0.16)	1.18 (0.18)	1.15 (0.17)
Observed ASVs	195.13 (52.25)	201.21 (54.40)	197.73 (53.26)
Shannon diversity	4.21 (0.36)	4.24 (0.37)	4.22 (0.36)
Simpson diversity	0.97 (0.02)	0.97 (0.02)	0.97 (0.02)

Data are mean (SD) or percentage. ABI, ankle-brachial index; AIX, aortic augmentation index; ASV, Amplicon Sequence Variants; BMI, body mass index; MAP, mean arterial pressure; PWV, pulse wave velocity;

We identified a total of 34,993 ASVs in the 3,087 samples. Shannon and Simpson indices of gut microbiota diversity were not significantly associated with VS, but we observed an increase of 0.10 (± 0.04) PWV m/s (p-value 0.02) and 0.6 (± 0.18) AIX units (p-value 0.01) per SD increase of observed number of ASVs (Table 2).

**Table 2:** Associations between microbiome diversity and continuous measures of vascular stiffness in the EPIC-DZD study.

Vascular stiffness	Beta coefficients*	SE	*P* value
PWV			
Shannon	0.02	0.04	0.57
Observed ASVs	0.10	0.04	0.02
Simpson	− 0.05	0.04	0.25
ABI			
Shannon	< 0.01	< 0.01	0.82
Observed ASVs	< 0.01	< 0.01	0.76
Simpson	< 0.01	< 0.01	0.56
AIX			
Shannon	0.34	0.18	0.06
Observed ASVs	0.60	0.18	0.001
Simpson	0.04	0.18	0.83

* per SD increase in microbiome traits.

ABI, ankle-brachial index; AIX, aortic augmentation index; ASV, Amplicon Sequence Variants; PWV, pulse wave velocity.

In addition, to verify associations of the whole microbiota composition with VS, we conducted a PERMANOVA analysis with the Euclidean distance matrix (on CLR transformed ASVs) against the VS parameters (adjusted for age, sex, BMI, MAP and batch) and the result did not show statistically significant associations (Table S1).

**Figure 1:**
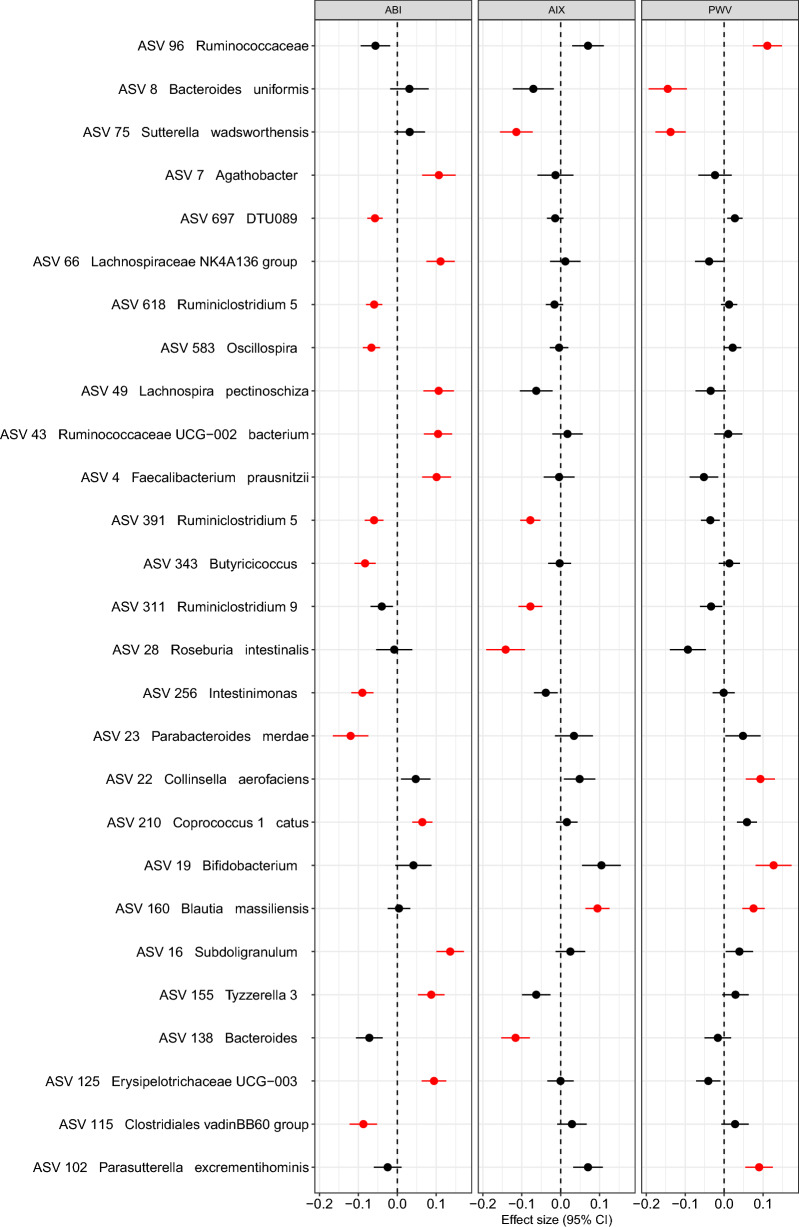
Effect size (coefficients) of standardized vascular stiffness measures associated with CLR transformed ASV counts (false discovery rate <  = 0.1 with at least 1 stiffness measure) adjusting for age, sex, BMI, MAP, batch, and sequencing run (Model A). ASVs in red have q-value <  = 0.1.

To further investigate individual ASVs associations, we tested 213 ASVs (present in at least 20% of the samples) using a multivariable linear model, finding a total of 19 ASVs associated with at least one VS parameter (model A, Fig. 1). Further adjusting by prevalent T2D (model B), 24 of those 27 ASV remained significantly associated (Figure S1). Three ASVs were statistically significantly associated (q-value < 0.1) with two VS measures. For example, we observed decreased CLR transformed counts of ASV 75 (classified as *Sutterella wadsworthensis*) with increasing AIX (beta = − 0.11 ± 0.04) and PWV (beta = − 0.14 ± 0.03). The CLR transformed count of the ASV 160, classified as *Blautia massiliensis*, was positively associated with AIX (beta = 0.09 ± 0.03) and PWV (0.08 ± 0.03). The ASV 391, classified as *Ruminiclostridium 5*, was negatively associated with AIX (− 0.08 ± 0.02) and ABI (− 0.06 ± 0.02). The other ASVs were not significantly associated with more than one VS measure after multiple test adjustments, but for most of them, their p-values were smaller than 0.05 and the directions of the association agreed (Fig. 1). In a sensitivity analysis, we further adjusted model B for current smoking status and found 19 ASVs remained significantly associated (Figure S2).

We investigated potential effect measure modification of the association between all ASVs and each VS measures by sex or prevalent T2D. From the ASVs that were significantly associated with VS on models A and B, we found just one significant interaction (ASV 7—*Agathobacter*) with T2D and no interaction with sex. However, for 5 other ASVs, the interaction analysis of VS and sex were significant (one with PWV, two with ABI and two with AIX). Regarding interaction with prevalent T2D, no significant interaction was observed in relation to PWV, however, 4 ASVs showed significant effect measure modification by type 2 diabetes status in their correlation with ABI and one in relation with AIX (Table S2). For those ASVs with significant interaction with prevalent T2D or sex, we conducted a stratified analysis, and the p-values and estimates are shown in Table S3.

## Discussion

In a cross-sectional analysis within the EPIC-Potsdam Study, we observed associations between different measures of VS (PWV, AIX and ABI) and individual microbiota components. We did not see significant associations of Shannon and Simpson alpha diversity indexes and PERMANOVA analysis of the distance matrix (beta-diversity) did not show significant association with any VS measures. Associations of VS with gut microbiota diversity remains controversial. A previous study in women from the Twins UK cohort^18^ found microbial diversity negatively associated with PWV when adjusted for possible confounders like age and MAP. On the other hand, a recent study found no associations of beta and alpha diversity indexes with VS^19^. However, that study was conducted with a small sample (69 participants) and the VS measure was determined by AASI.

The lack of a significant association of VS with gut microbiome diversity is somehow unexpected because dysbiosis (decreased diversity in the microbiota) has been associated with several diseases, including cardiovascular conditions^10,25^. One possible explanation for those inconsistencies between our study and the one conducted on UK twins might relate to differences in age between the two studies (68.6 compared to 61.4 years^18^), and it was demonstrated that gut microbiome diversity decreases with age, even in healthy individuals^26^. In addition, the previous cohort included only female twins, had fewer cases of prevalent T2D, and measured only PWV.

We furthermore investigated individual components of the microbiota for association with VS. The inverse association of an ASV classified as *Sutterella wadsworthensis* with PWV and AIX suggests a protective effect. *S. wadsworthensis* is a bile-resistant species that was associated positively and negatively with several health conditions^27^. More importantly, a study found positive association of *Sutterella* genus with blood pressure^7^ and stroke^28^, while another study found negative association with heart failure^29^ and obesity^30^. One possible reason for the positive effect of *S. wadsworthensis* on VS might be a suggested immunomodulatory function of this bacterium^27^, however, a more accurate functional prediction is impossible without further experiments. None of the previous two studies on VS and gut microbiome composition observed a significant correlation of *Sutterella* abundance and VS, however one species not detected in our study—*Sutterella timonensis*—correlated to a higher VS index^19^. In the same study, the levels of plasma SCFA were negatively correlated with VS, but also could not be attributed to the gut microbiota composition. SCFA concentrations were not investigated in our study, but the microbiome composition was not shifted to more SCFA-producing genera (mainly of Firmicutes), and *Sutterella* is a bacterial genus not characterised as SCFA-producing. Remarkably, the ASV classified as *Ruminiclostridium* 5, containing known SCFA-producers^31^, and the HDL-C enhancing *Eubacterium siraeum*^32^ were negatively correlated to AIB and AIX. In contrast, *Blautia massiliensis*, positively correlating with AIX and PWV, was recently detected to positively correlate with high systolic blood pressure^33^.

The observation that a Ruminococcaceae and one *Colinsella aerofaciens* ASV were positively associated with PWV is not consistent with the study in the TwinsUK cohort that found OTUs of those taxa negatively associated with PWV^18^. The Ruminococcaceae is a large family including beneficial microorganisms (e.g. *Faecalibacterium prausnitzii*) as well as bacteria possibly contributing to higher TMA and/or TMAO levels^34,35^, which are choline metabolites that are an independent risk factor for promoting atherosclerosis^36,37^. Unfortunately, the 16S rRNA gene sequences of Ruminococcaceae did not allow a more exact classification in our and the previous study making the results difficult to compare. *Collinsella aerofaciens* was positively associated with cardiac valve calcification risk in T2D patients^38^, individuals with CAD risk^39^, and with pulmonary arterial hypertension^40^. Recently, *C. aerofaciens* was found to be decreased in individuals following a Mediterranean diet compared with those following a non-Mediterranean diet, and it was hypothesised that this could be a potential mediator effect of the benefits of this diet^41^. It is important to note that in model B (adjusted for prevalent T2D), *C. aerofaciens* did not remain significantly associated with PWV and this might indicate that this correlation is cofounded by prevalent T2D and/or treatment.

When compared to Menni et al.^18^, divergences in the current study can be due to differences among cohorts, but some different results might be explained by the microbiota analysis method itself. For example, we sequenced the longer V3-V4 region of the 16S rRNA gene instead of only the V4 region, leading to a better taxonomic resolution and a more accurate classification^42^. In addition, the sequences were processed with DADA2 for generating ASVs, keeping individual sequence resolution instead of the operational taxonomic unit (OTU) approach which groups sequences based on an arbitrary sequence similarity cutoff. The latter approach may have detrimental effects in biomarker screening studies as it potentially clusters together different bacterial species that might have contradictory effects on the targeted outcome^43^. We also used more appropriate methods for analysing compositional data, transforming the read counts using the centre-log ratio (CLR) approach before proceeding to linear models for association analysis. It was recently demonstrated that using standard normalisation methods for microbiome abundance data leads to a negative correlation bias^44^, and to avoid this issue the data should be addressed as a composition. Contrary to our study, Menni et al.^18^ used relative abundance normalised by total read counts, which might explain why they found only significantly negative associations.

Interestingly, we observed a larger number of ASVs significantly associated with ABI (17 ASVs) compared to PWV (7), and contrary to PWV, most of the ASVs associated with ABI suggested a beneficial effect (9 positively associated). ABI is a ratio of systolic blood pressure measured at the ankle to the brachial artery. A low value (ABI =  < 0.9) is indicative of PAD and it was shown that it is also an indicator of atherosclerosis at other vascular sites^45^. Our results suggest that different components of the microbiota might affect central and peripheral VS differently due to the small overlap of significantly associated ASVs with PWV and ABI.

Some of the ASVs that are associated with lower VS were classified as species currently regarded as probiotics. For example, *Bacteroides uniformis*, a butyrate-producing bacterium generally associated with good health conditions^46–48^, was negatively associated with PWV. A strain of *B. uniformis* (CECT 7771) isolated from the gut microbiota of infants has been tested as a probiotic for safety in prolonged usage in mice and rats^48^ and it is being considered for clinical studies in humans^49^. It was also demonstrated in mice that this strain can ameliorate impaired glucose intolerance^46^. Another example of a potential probiotic is an ASV classified as *Faecalibacterium prausnitzii*, positively associated with ABI. However, in model B, further adjusted by prevalent T2D, this ASV was not significantly associated with ABI. This species, a core member of the healthy gut microbiota^50^, is one of the main butyrate-producing bacteria in the gut, being found inversely associated with several conditions, such as obesity^51^, T2D^1^, hypertension^6,7^ and heart failure^25^. Important to note is that some specific functions of probiotic bacteria are strain-specific and cannot be resolved in a 16S rRNA gene-based study.

Our study has further limitations. Even though a longer 16S rRNA gene region was sequenced compared to the TwinsUK cohort study^18^, the sequence is still partial and therefore an exact taxonomic classification of the bacteria was not possible. In addition, the focus on differences in the abundance of individual strains neglects that the gut microbiome function is based on an interplay of different strains, which cannot be assessed in this study. The genetic repertoire of the microbiome can only be revealed by whole metagenome sequencing, which should be performed in future studies. Similarly, further studies should assess if dietary factors, which were not available in this study, affect or explain the observed associations. Faecal sampling was not performed under controlled conditions, possibly influencing the microbiome analysis. However, due to the large cohort analysed in this study, faecal sampling under controlled conditions is not feasible. Furthermore, we could not assess a potential connection between microbiome and vascular stiffness measures via inflammation as inflammatory biomarkers were not available in this study sample.

To conclude, in an EPIC-Potsdam subsample, we found cross-sectional evidence for associations of several taxa with VS measures, but only limited evidence for associations of indicators of microbial diversity with VS. The study sample size, the inclusion of men, the inclusion of additional VS measures, and application of state-of-the-art bioinformatic analysis techniques led our study to challenge previous findings.

### Supplementary Information

Supplementary Figure S1.

Supplementary Figure S2.

Supplementary Table S1.

Supplementary Table S2.

Supplementary Table S3.

## Data Availability

The datasets analysed during the current study are not publicly available due to data protection regulations. In accordance with German Federal and State data protection regulations, epidemiological data analyses of EPIC-DZD may be initiated upon an informal inquiry addressed to the secretariat of the Human Study Center (Office.HSZ@dife.de). Each request will then have to pass a formal process of application and review by the respective PI and a scientific board.
