# Supplementary material for: Association of the human gut microbiota with vascular stiffness
Source: Sci Rep. 2023 Aug 16;13:13348. doi: 10.1038/s41598-023-40178-6 (PMC10432492; doi:10.1038/s41598-023-40178-6)

Figure S2: Effect size (coefficients) of standardised vascular stiffness measures associated with CLR transformed ASVs abundances (false discovery rate < = 0.1 with at least 1 VS measure) adjusting for age, sex, BMI, MAP, batch, sequencing run, diabetes status and smoking status (Model C). ASVs in red have q-value <= 0.1.

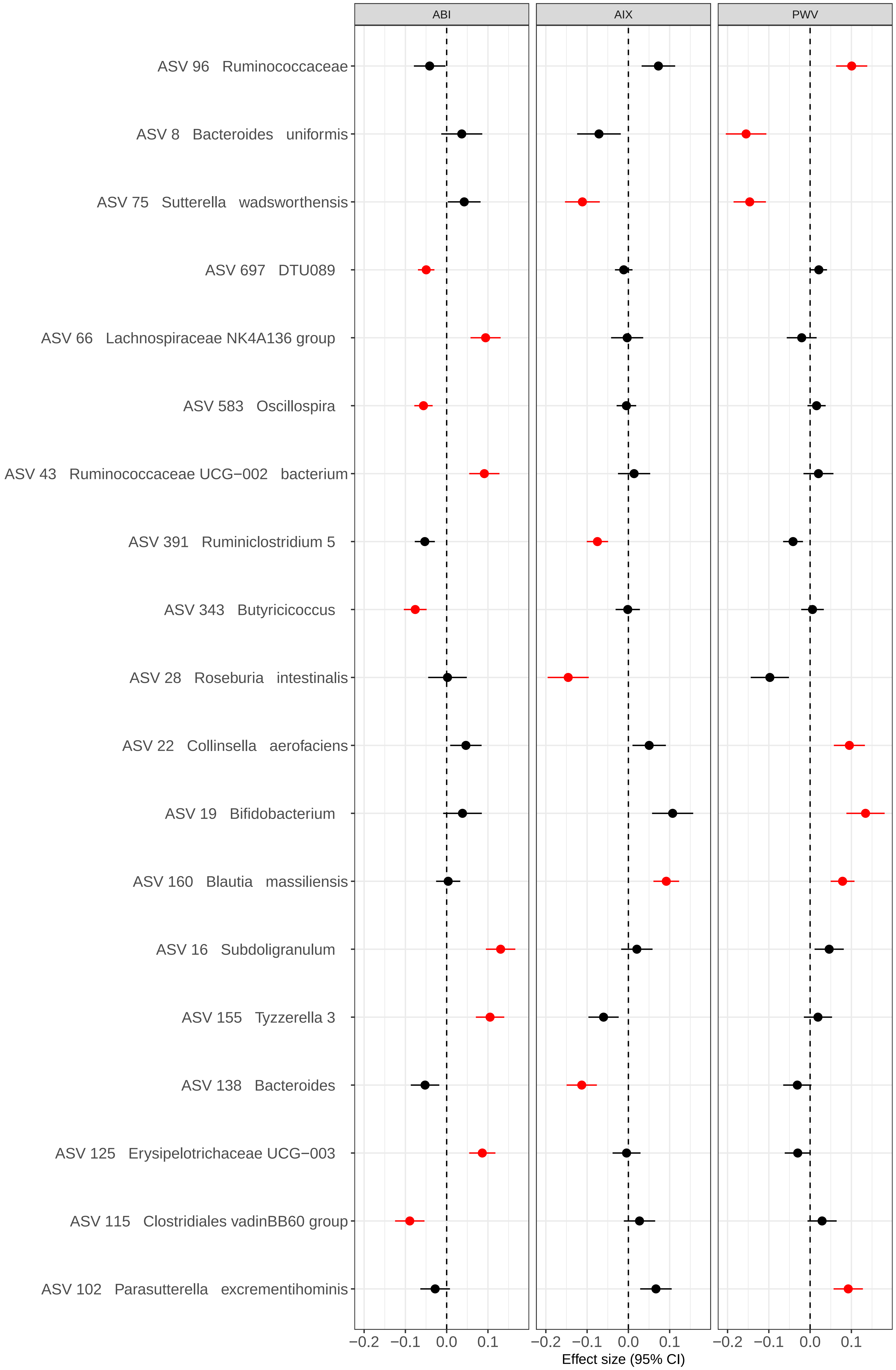

Supplement: Supplementary file 2 — Supplementary Figure S2. [file 41598_2023_40178_MOESM2_ESM.pdf]
